# 

**Published:** 1892-01

**Authors:** 


### CONTENTS.

Original Articles—Croupous Pneumonia—By R. M. Cunningham, M. D., Birmingham, Ala....1
The Cure of Epithelioma—By W. S. Gottheil, M. D., New York....16
Malarial Hæmoglobinuria—By H. McHatton, M. D., Macon, Ga....21
Correspondence—Our New York Letter....26
Society Notes—Southern Surgical and Gynæcological Society of Baltimore....38
Editorials—Death of Dr. Henry Campbell....41
Editorial Notes....43
The East Tennessee, Virginia & Georgia Tariff....45
Announcement....46
Book Reviews—Syllabus of Obstetrical Lectures....48
The Medical Review Visiting List Perpetual....48
The Medical News Visiting List for 1892....48
Blakiston's Visiting List for 1892....49
Selections and Abstracts—The Treatment of Syphilis....50
Specific Abortive Treatment of Pneumonia....51
The Anal Reflex....52
Treatment of Influenza with Camphor....53
Trephining in a Case of General Paralysis....53
Gonorrhœa of the Mucous Membrane of the Mouth of the New-Born..54
Gunpowder Stains....54
Acute Rheumatism Confined to the Temporo-Maxillary Joint....55
Treatment of Infantile Paralysis....47
Death From Anæsthetics....29
Bassinis Radical Cure of Hernia....25
Prescription Department....60–61
LaGrippe—Syrup for Infantile Constipation—Bilious Headache—Nocturnal Emissions—Poison Oak Eruptions—Injection for Gonorrhœa—Tonic and Laxative—Cardiac Debility.
Special Notes....56–59
Fellows' Hypophosphites—Bromidia—Mennon's Borated Talcum Powder—Atmospheric Tractor—Quickine in Croup.—Neurosine in Epilepsy and Chorea—Terraline—Reed & Carnrick—Sanders & Sons' Eucalyptol Extract—Listerine—The Empire Abdominal Supporter.
Index to Vol. XXI....1 to 5

